# Translation and Validation of the Thai Version of the Nasal Obstruction Symptom Evaluation (NOSE) Scale

**DOI:** 10.1002/oto2.29

**Published:** 2023-03-01

**Authors:** Kanokpon Prasertwit, Kangsadarn Tanjararak, Navarat Tangbumrungtham, Alongkot Emasithi, Boonsam Roongpuvapaht

**Affiliations:** ^1^ Department of Otolaryngology, Faculty of Medicine Ramathibodi Hospital Mahidol University Bangkok Thailand

**Keywords:** deviated nasal septum, nasal airway obstruction, NOSE, validation

## Abstract

**Objective:**

The Nasal Obstruction Symptom Evaluation (NOSE) was developed to evaluate subjective outcomes of patients with deviated nasal septum and symptomatic nasal obstruction. Considering the differences in individuals' cultural, cross‐cultural translation, adaptation, and validation of the instrument are necessary. The current study aimed to translate and validate the Thai version of the NOSE Questionnaire for patients with nasal septum deviation.

**Study Design:**

A single‐center prospective instrument validation study.

**Setting:**

Thai tertiary referral center.

**Methods:**

The study was conducted to translate and adapt the original English version of the NOSE to Thai. After translating, psychometric testing was conducted. The primary outcomes were validity (content, construct, and discriminant), reproducibility (test‐retest procedure), and internal consistency (reliability). A total of 105 participants, of which 46 were patients with nasal airway obstruction and 59 were healthy asymptomatic volunteers, were enrolled in this study.

**Results:**

The Thai‐NOSE was found to be adequate for all tested psychometric properties with high internal consistency (Cronbach's *α* = .942), and to discriminate accurately between patients and healthy controls. The interitem and item‐total correlations indicated a related construct among all items. A high level of reproducibility of the questionnaire was obtained in the test‐retest procedure for each item (*γ* = 0.898). The initial test and retest scores indicated adequate reproducibility.

**Conclusion:**

The Thai‐NOSE questionnaire is a reliable instrument with appropriate psychometric properties for assessing the severity and impact of nasal airway obstruction in patients with nasal septum deviation.

Nasal airway obstruction is one of the most common symptoms among patients visiting otolaryngology outpatient clinics.^1^, ^2^, ^3^ The complexity of this symptom is composed of multifactorial etiologies including individual anatomical structure, inflammatory disease, and compounding physiological factors.^4^, ^5^ Nasal obstruction induces a feeling of blockage and impedes the air flow through the nose, and it can have a dramatic impact on patients' quality of life.^6^, ^7^, ^8^ Presently, there is no standard diagnostic algorithm for assessing and evaluating nasal airway obstruction and posttreatment change.^9^

Many diseases involve this symptom, including inferior turbinate hypertrophy, acute and chronic rhinosinusitis, nasal polyposis, allergic rhinitis, nasal valve compromise, and deviated nasal septum (DNS).^3^, ^4^, ^6^, ^7^, ^10^, ^11^ DNS is one of the most common etiologies, and, in addition to nasal airway obstruction, causes other symptoms such as epistaxis, headache, obstructive sleep apnea, and recurrent sinusitis.^12^, ^13^, ^14^

There are many objective measurements for evaluating nasal patency. However, in most cases, the severity of nasal airway obstruction is not correlated with objective physical findings.^5^, ^15^ The Nasal Obstruction Symptom Evaluation (NOSE) scale, developed by Stewart et al,^11^ is the first subjective clinical report and disease‐specific quality of life instrument for evaluating the symptoms of nasal airway obstruction in patients with DNS.

Although there are many methods for evaluating objective outcomes, such as acoustic rhinometry, rhinomanometry, and computed tomography, there are still discordant and limited correlations between these objective assessments and patient symptoms.^1^, ^4^, ^7^, ^16^, ^17^ In addition to patient symptoms, well‐validated instruments are considered to provide an appropriate assessment of the outcomes of surgery.^15^ Furthermore, many other validated disease‐specific quality‐of‐life instruments have been developed, such as the Sino‐Nasal Outcome Test‐22 (SNOT‐22) and the Chronic Sinusitis Survey (CSS); however, none of these instruments are specifically designed to evaluate nasal obstruction symptoms.^6^, ^11^ Consequently, in 2004, the NOSE (Figure 1) was used as a valuable instrument for evaluating subjective outcomes of patients with DNS and symptomatic nasal obstruction.^11^ The NOSE is composed of 5 items that are related to nasal obstruction symptoms. Each item is scored from 0 to 4 using a 5‐point Likert scale.^7^, ^11^

**Figure 1:**
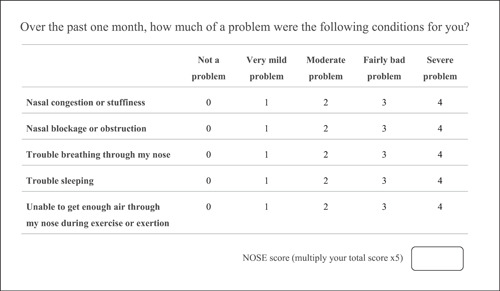
Items of Nasal Obstruction Symptom Evaluation (NOSE).

Unlike the other questionnaires used in rhinological practice, the NOSE is a disease‐specific instrument that is brief, easily self‐completed, and less burdensome for respondents.^6^, ^7^, ^11^ Moreover, this questionnaire is specific to nasal obstruction and can be used to assess health status before and after operative treatment.^3^, ^11^ Therefore, the NOSE is beneficial not only for following up patients with ongoing medical treatment but also for evaluating patients after definite treatment.^1^, ^6^, ^17^ NOSE scores are reported to be correlated with nasal septal deviation severity graded by physicians, objective computed tomography (CT) findings,^1^ and the results of a validated instrument for septoplasty and functional rhinoplasty.^17^ According to the original study and subsequent validation studies of the NOSE, the instrument exhibits good internal consistency, reliability, validity, stability, and sensitivity.^3^, ^8^, ^11^ Because of these benefits, the NOSE was translated into many other languages, including Greek,^2^ Portuguese,^7^ Turkish,^8^, ^18^ Spanish,^19^ French,^20^ Polish,^21^ Italian,^22^ German,^23^ Dutch,^24^ and Lithuanian.^25^

Because of the multidimensional concepts of health, assessment of personal well‐being cannot be limited only to physical health, but must also include quality of life in a holistic care approach.^2^, ^22^ Considering individual differences in culture, linguistic, and ethnic background, forward translation of the instrument is insufficient.^26^ Thus, cross‐cultural translation, adaptation, and validation of the instrument are necessary.^26^ Translated and adapted instruments need to be evaluated for validity, reliability, internal consistency, and response sensitivity before being used in a target population.^26^

In 2012, Sousa et al^26^ reviewed the recommended methodologies for cross‐cultural translation, adaptation, and validation of a healthcare‐related instrument. According to these recommendations, 7 steps should be followed: (1) forward translation, (2) comparison of 2 translated versions, (3) backward translation, (4) comparison of 2 backward translated versions, (5) pilot testing of prefinal version, (6) preliminary psychometric testing, and (7) full psychometric testing.^26^

The NOSE has not previously been translated and adapted to the Thai population. In the current study, we developed a Thai translation and adaptation of the NOSE questionnaire in patients with DNS.

## Materials and Methods

### Study Design

This study was a single‐center prospective instrument validation study conducted in the otolaryngology outpatient clinic at Ramathibodi Hospital (tertiary referral center). The protocol was approved by the medical ethics committee of the Ramathibodi Hospital, Mahidol University (COA.MURA2021/317). All data were prospectively collected from April 2021 to January 31, 2022. Two phases of this study included cross‐cultural item translation and adaptation, and psychometric testing.

### Phase I: Cross‐Cultural Item Translation and Adaptation

**Figure 2:**
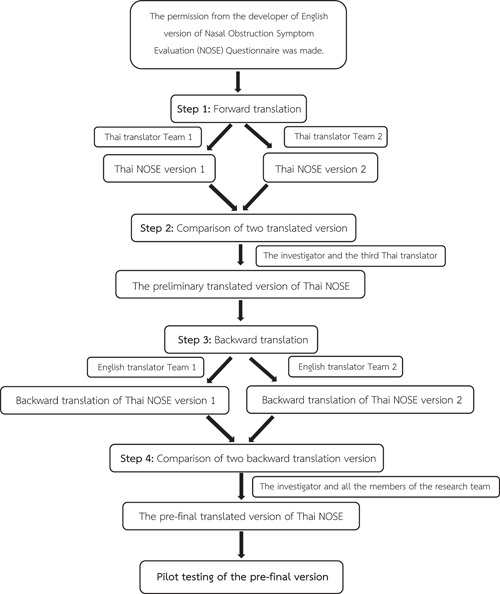
Protocol flow chart.

To provide a reliable and validated cross‐cultural instrument, the translation and validation protocol (Figure 2) strictly followed the methodological guidelines for cross‐cultural health care research published by Sousa et al.^26^

After permission was obtained from the developer of the original English version of the NOSE questionnaire, forward translation from the original version to create the Thai‐NOSE version was performed by 2 independent, bilingual and bicultural native Thai‐speaking translators. One of the translators was familiar with medical and healthcare terminology. Subsequently, all investigators met together with a third independent Thai translator, discussed the wording, adjusted the clarity of the language, and merged the 2 translated versions of the questionnaire into a preliminary version of the Thai‐NOSE.

The preliminary version of the Thai‐NOSE was then translated back into English (blind backward translation) by 2 independent, bilingual and bicultural native English‐speaking translators. Again, 2 backward translated versions of the questionnaire were discussed, compared, and resolved by all of the investigators (composed of 3 rhinologists and 1 methodologist). The investigators also discussed the translation with Dr Stewart, the developer of the original version of the instrument, to clarify discrepancies in wording and to obtain permission to add details describing each item of the Thai‐NOSE. These details were intended to clarify some ambiguous wording in the Thai version. Content validity was verified during this phase. Finally, after resolving all of these ambiguities, the prefinal version of the Thai‐NOSE was created.

A pilot study of the prefinal version of the Thai‐NOSE was conducted with 10 Thai native individuals who were general and subspecialty staff in otolaryngology, and fellows and residents in the otolaryngology department at Ramathibodi Hospital to rate the clarity of the questionnaire.

**Figure 3:**
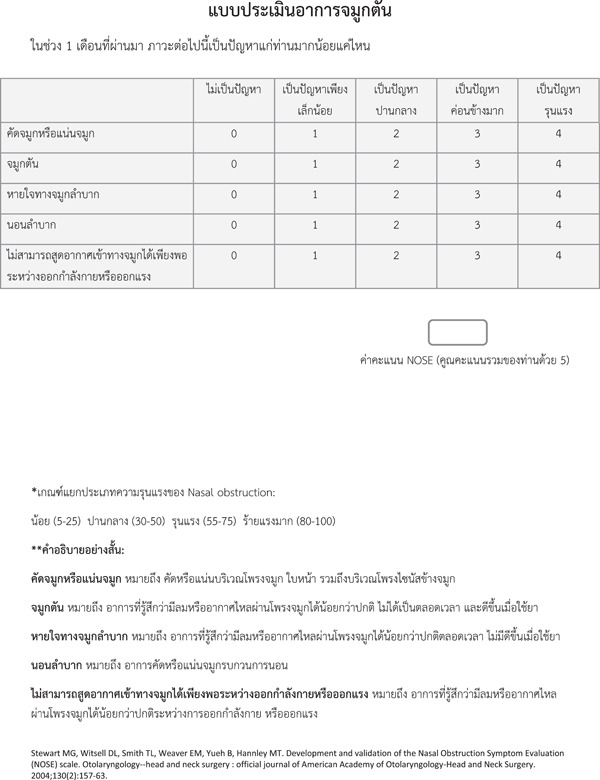
Final version of Thai‐NOSE. NOSE, Nose Obstruction Symptom Evaluation.

Preliminary psychometric testing of the prefinal version was conducted using 5 subjects per instrument item (total of 25 subjects). These subjects comprised healthy volunteers, residents from other departments and medical staff other than otolaryngologists. Data were collected for discussion and comparison and used to resolve any ambiguity in the instrument. When all ambiguity was resolved, the end result was the final version of the Thai‐NOSE (Figure 3).

### Phase II: Psychometric Properties Testing

#### Study Populations and Procedure

In accordance with the original study and related previous studies, internal consistency reliability was calculated using Cronbach's *α* coefficient, in which an *α* ≥ .70 was considered to be satisfactory.^7^, ^11^ The upper limit of the population correlation was set to .85, and the lower limit was set to .70. The number of participants required for the study was determined to be 105 (n = 105).

To validate the instrument, we recruited 2 groups of participants. The first group consisted of patients who were able to read and speak Thai, were aged at least 18 years old, who had nasal septal deviation with clinical chronic nasal airway obstruction lasting at least 3 months, and who had persistent symptoms after 4 weeks of medication (tropical nasal steroid, decongestants, oral antihistamine). The diagnosis was performed using rigid endoscopy or CT scans. Patients in this group were recruited when they visited the otolaryngology outpatient clinic at Ramathibodi Hospital. All patients with sinonasal malignancy, previous or ongoing radiation therapy of the head and neck, previous septoplasty, rhinoplasty or turbinoplasty, a history or clinical evidence of chronic sinusitis, nasal septal perforation, craniofacial syndrome, nasal trauma or fracture in the past 3 months, nasal valve collapse, adenoid hypertrophy, sarcoidosis, Wegener's granulomatosis, uncontrolled asthma, or pregnancy were excluded from this study. The second group were healthy asymptomatic volunteers who were aged over 18 years old and able to read and speak Thai. Those who met the criteria and provided written informed consent were asked to complete the Thai‐NOSE by themselves at baseline and repeat it again 14 days later to assess test‐retest reliability.

#### Outcome Measurements

The primary outcomes in this study were validity (content, construct, and discriminant), reproducibility (test‐retest procedure), and internal consistency (reliability).

#### Statistical Analysis

The baseline demographic data were compared between the 2 groups and presented as mean ± standard deviation and percentage values. Content validity was ensured during the instrumental translation protocol. Construct validity was assessed using inter‐item and item‐total Spearman's correlation coefficients, and a coefficient of ≥0.40 was set as the significance level of associations.^11^, ^20^ Discriminant validity (between‐group discrimination) was assessed by comparing total scores of each item between patients and a sample of healthy volunteers. The difference between the Thai‐NOSE scores in the patient and control groups was then analyzed using an independent *t* test. The reproducibility of the questionnaire was assessed from the mean total scores obtained in the test‐retest procedure using the Goodman‐Kruskal *γ* coefficient, with a value of at least 0.70 being considered adequate.^11^ Moreover, the paired *t* test was used to compare the Thai‐NOSE score between the initial test and the retest of each item after 14 days. Internal consistency (reliability) of the Thai‐NOSE was evaluated using Cronbach's *α* coefficient; an *α* ≥ .70 was considered adequate.^11^ All statistical analyses were conducted with STATA 17. The level of statistical significance was set at *p* < .05.

## Results

### Validity

After cross‐cultural translation and adaptation, the Thai version of the NOSE (Thai‐NOSE) was created. During translation following the recommended methodology, content validity was ensured. A total of 105 participants, of which 46 were patients with nasal airway obstruction and 59 were healthy asymptomatic volunteers, were enrolled in this study. The mean ages were 42.96 ± 14.89 (23‐73) years for the patients in the nasal airway obstruction group and 33.08 ± 8.91 (21‐60) years for the control group of healthy asymptomatic volunteers. The baseline demographic data of both groups are shown in Table 1. All of the subjects in both groups completed the questionnaire without any assistance at 2 different times, during the first enrollment visit (as initial test) and 14 days later (retest).

**Table 1:** Demographic Data

	Nasal airway obstruction (n = 46)	Control (n = 59)
Age	42.96 ± 14.89	33.08 ± 8.91
Sex		
Male	23 (50%)	7 (11.9%)
Female	23 (50%)	52 (88.1%)

### Construct Validity

Inter‐item and item‐total Spearman's correlation coefficients (Spearman's *ρ*: *r*) are reported in Table 2, using data from 46 patients with DNS and nasal airway obstruction. A significant level of association was set as a coefficient (*r*) of ≥0.40. The expected associations of inter‐item and item‐total correlations were consistent with the prior original English version and previously validated translated versions.^11^, ^20^, ^24^, ^25^ Additionally, the translated instrument illustrated a related construct among all items, but not all of the items used the same concept of measurement. A significant correlation was found for most of the items, but not for the correlation between “Nasal congestion or stuffiness” and “Unable to get enough air through my nose during exercise or exertion” (*r* = 0.356, *p* = .015). However, in a comparison of item‐total correlations, all items exhibited high correlation coefficients with total test scores.

**Table 2:** Item‐Item Correlations for the 5 Items of the Thai‐NOSE (Nasal Airway Obstruction n = 46)

	Nasal congestion or stuffiness		Nasal blockage or obstruction		Trouble breathing through my nose		Trouble sleeping		Unable to get enough air through my nose during exercise or exertion	
	*r*	*p*	*r*	*p*	*r*	*p*	*r*	*p*	*r*	*p*
Nasal blockage or obstruction	0.514	<.001	1	‐						
Trouble breathing through my nose	0.676	<.001	0.628	<.001	1	‐				
Trouble sleeping	0.489	.001	0.688	<.001	0.68	<.001	1	‐		
Unable to get enough air through my nose during exercise or exertion	0.356	.015	0.427	.003	0.471	.001	0.502	<.001	1	‐
Total score	0.706	<.001	0.825	<.001	0.862	<.001	0.84	<.001	0.7	<.001

Spearman's *ρ* (*r*).

### Discriminant Validity

Comparing total scores of each item between study patients and control group, the independent *t* test was used with an expected significant level of difference defined as *p* < .05. The instrument was found to perform very well at discrimination for all items and the total score, and this is shown in the last column of Table 3.

**Table 3:** Test‐Retest Reliability Analysis in Patients With Nasal Airway Obstruction (n = 46) and the Control Group (n = 59)

	Nasal obstruction (n = 46)			Control (n = 59)			*p* value
	Test	Retest	*p* value^a^	Test	Retest	*p* value^a^	Between group
Nasal congestion or stuffiness	1.93 ± 0.98	1.96 ± 0.94	.710	0.63 ± 0.64	0.51 ± 0.57	.109	<.001
Nasal blockage or obstruction	1.91 ± 1.07	1.78 ± 1.05	.032	0.41 ± 0.59	0.42 ± 0.59	.811	<.001
Trouble breathing through my nose	1.48 ± 1.07	1.43 ± 1.07	.420	0.25 ± 0.51	0.22 ± 0.49	.621	<.001
Trouble sleeping	1.43 ± 1.17	1.5 ± 1.09	.261	0.24 ± 0.47	0.17 ± 0.38	.252	<.001
Unable to get enough air through my nose during exercise or exertion	1.13 ± 1.09	1.2 ± 1.11	.183	0.31 ± 0.68	0.24 ± 0.6	.321	<.001
Total score	7.89 ± 4.34	7.87 ± 4.35	.888	1.83 ± 2.28	1.56 ± 2.00	.295	<.001

^a^ Paired *t* test.

### Internal Consistency (Reliability)

Compared with the original English version, higher correlations were found in this study. The Cronbach's *α* coefficients were .942 and .785 for the Thai‐NOSE and original English versions, respectively. This coefficient represented the precision and homogeneity of the instrument.

### Test‐Retest Reliability

Data for test‐retest evaluation were accepted for all 105 patients in both groups. Scores from the initial test and retest of both groups were calculated separately. The reproducibility of the questionnaire was assessed from the mean total scores obtained in the test‐retest procedure of each item using the Goodman‐Kruskal *γ* coefficient and was found to be very good, with *γ* = 0.898.

Furthermore, paired *t* tests were used to compare the Thai‐NOSE score between the initial test and the retest of each item, as shown in Table 3. Only 1 item, nasal blockage or obstruction (*p* = .032) in the nasal airway obstruction group, did not meet the significance level. Instead, this item in the control group reached a significant level of reproducibility (*p* = .811). The data mentioned above indicated that all items had adequate reproducibility.

## Discussion

Nasal airway obstruction has multifactorial etiology, and can interfere with the perceived quality of life of patients. Many objective and subjective measurements have been developed to assess the degree of severity and outcomes of treatment.^9^, ^27^ Moreover, evaluation using diagnostic algorithms for nasal airway obstruction has also been attempted, although there are difficulties in the application of this algorithm because of the diversity of anatomical causes, discordance between objective examination and subjective perception of patients, and variability among examiners.^5^, ^9^ As a result, no standardized classification or algorithm has been specifically applied to this symptom.^27^ Subjective reported outcomes have received more attention in the assessment of nasal airway obstruction and therapeutic success.^27^, ^28^ A systematic review and meta‐analysis conducted in 2017 focused on evaluating the outcomes of nasal airway surgery using NOSE scores.^29^ The results revealed that the improvement of nasal obstruction measured by the NOSE after functional rhinoplasty was sustained for more than 12 months.^29^ Significant improvement in patients following functional septorhinoplasty was observed as early as 1 to 3 months.^29^, ^30^

To ensure the equivalence between the original version of the NOSE and the Thai‐NOSE, and to maintain the concepts of the original instrument, cross‐cultural translation, adaptation, and validation were conducted. Considering variation in culture, language, and ethnic background, the translation protocol was conducted following the methodological guidelines.^26^ One difficulty in translating the original instrument was the similarity of meaning of translated words in Thai, which could potentially be confusing for patients. To minimize this problem, some details describing each item of the Thai‐NOSE were added to the end of the questionnaire. These details were added after obtaining permission from the developer of the original instrument. Moreover, all of these descriptions were implemented under the supervision of the developer to ensure equivalence of the concepts of measurement compared with the original version. None of the items had a high inter‐item correlation (*r* ≥ 0.7), which suggested the excessiveness of the items in the instrument.

The Thai‐NOSE instrument had satisfactory psychometric properties and was consistent with the original English version. The reliability coefficient was found to be very good, with *γ* = 0.898 in this study (*γ* = 0.702 for the original English version).^11^ When comparing this coefficient with other validated studies, the *γ* coefficient for the Thai‐NOSE was higher than the coefficient of the Portuguese version (*γ* = 0.776) but lower than the coefficient of the Spanish version (*γ* = 0.962).^7^, ^19^ Thus, the overall *γ* coefficients of these studies were found to have adequate properties, with a value of at least 0.70.^11^ Cronbach's *α*, which represents internal consistency, was found to be satisfactory with *α* = .942, comparable with the original study (*α* = .785) and previous studies (*α* up to .955 in the Spanish version).^11^, ^19^, ^23^, ^25^ The Goodman‐Kruskal *γ* coefficient and Cronbach's *α* coefficient reported in this study were calculated from the data of both groups (nasal airway obstruction patients and healthy controls) to verify the adequacy and efficiency of the instrument in both populations. Furthermore, the translated instrument appeared to be a sensitive and valuable tool for discriminating between patients with the disease and the healthy population.

This validated instrument not only aims to assess the severity of nasal airway obstruction but also the clinical outcomes after therapeutic approaches. Besides septoplasty, there are many other interventions (both surgical and medical) that can be evaluated using the NOSE questionnaire, such as intranasal steroid, radiofrequency ablation, and functional septorhinoplasty.^14^, ^30^, ^31^, ^32^ The Thai‐NOSE appears to be helpful for documentation and discrimination of patients suffering from various degrees of nasal airway obstruction. However, the NOSE is limited only to subjective clinical reports of nasal airway obstruction. Accurate diagnosis, which can determine the appropriate treatment options, cannot be made solely using this instrument. Hence, subjective evaluation with the Thai‐NOSE can be used in combination with objective rhinological data or other validated instruments to improve the accuracy of diagnosis, the impact of the disease on patients' quality of life, and therapeutic success.

The perception of disease or changes in each symptom may differ between patients. Importantly, the subjective instrument examined in the current study was able to detect and represent the patient's perception individually and present it as a numerical score, not just as descriptive data. As a result, the progression or change in clinical illness can be easily communicated between the patients and attending physicians.

The limitations of this study include a lack of data to indicate patients' responsiveness to therapeutic interventions, and the sampling of data without comparison with other validated and translated subjective instruments in Thai. Further prospective studies applying this validated instrument to assess the quality of life of patients suffering from diseases other than nasal septal deviation, the responsiveness of each therapeutic modality, and comparison of this instrument with objective data may provide further valuable findings.

## Conclusion

Following recommended methodologies, the Thai‐NOSE was developed, adapted, and validated, and was found to be reliable, with appropriate psychometric properties. The parameters demonstrated equivalence in validity (content, construct, and discriminant), reliability, and reproducibility when compared with the original English version. The Thai‐NOSE questionnaire is an appropriate instrument for assessing the severity and impact of nasal airway obstruction, and is able to indicate clinical outcomes after different therapeutic modalities.

## Author Contributions

**Kanokpon Prasertwit**, design, data collection, analysis and interpretation, drafting and revision of the manuscript; **Kangsadarn Tanjararak**, conception and design, interpretation; **Navarat Tangbumrungtham**, conception and design, interpretation; **Alongkot Emasithi**, translation, revision of the manuscript; **Boonsam Roongpuvapaht**, conception and design, acquisition of data, analysis and interpretation, revision of the manuscript.

## Disclosures

### Competing interests

None.

### Sponsorships

None.

### Funding source

None.
